# Heritability and Genetic Parameters for Semen Traits in Australian Sheep

**DOI:** 10.3390/ani12212946

**Published:** 2022-10-26

**Authors:** Marnie J. Hodge, Sally J. Rindfleish, Sara de las Heras-Saldana, Cyril P. Stephen, Sameer D. Pant

**Affiliations:** 1 School of Agriculture, Environment and Veterinary Sciences, Charles Sturt University, Wagga Wagga, NSW 2678, Australia; 2 Apiam Animal Health, Apiam Genetic Services, Dubbo, NSW 2830, Australia; 3 Animal Genetics and Breeding Unit, University of New England, Armidale, NSW 2351, Australia; 4 Gulbali Institute, Charles Sturt University, Boorooma Street, Wagga Wagga, NSW 2678, Australia

**Keywords:** genetic correlation, ram, ovine, ejaculate quality, repeatability

## Abstract

**Simple Summary:**

Semen traits associated with ejaculate volume, gross motility, concentration, and percent post-thaw motility are crucial determinants of successful reproduction, which, in turn, can influence the profitability of seedstock and commercial sheep enterprises. Previous studies in cattle, where artificial insemination is also widely used, have reported declining trends in ejaculate traits. Such a trend, if observed in sheep, could negatively impact conception outcomes, and contribute to reduced reproductive efficiency. Selective breeding to improve ejaculate traits could provide a means to ensure continued efficiency in ovine reproduction. Therefore, the objective of this study was to estimate heritability and genetic correlations between four routinely assessed ejaculate quality and quantity traits in sheep breeds commonly farmed in Australia. An analysis of ejaculate traits collected over 20 years indicated that all ejaculate quality and quantity traits were lowly heritable (0.081–0.17), with gross motility and volume having relatively higher heritability estimates compared to concentration and percent post-thaw motility. Overall, the results indicate that ejaculate quality and quantity could potentially be improved via selective breeding.

**Abstract:**

Semen characteristics including volume, gross motility, spermatozoal concentration, and percent post-thaw motility are routinely assessed to determine the quality and quantity of an ejaculate prior to use in artificial breeding programs. Currently, artificial breeding programs in sheep place relatively little emphasis on ram-side factors, such as the fertilising potential of an ejaculate, which may contribute to variability in conception outcomes. Estimating genetic parameters for ejaculate quality and quantity traits could provide insights into whether selective breeding can be used to improve such ram-side traits, improving ovine reproductive performance and farm profitability. Therefore, in this study, a total of 11,470 ejaculate records, including data for ejaculate volume, gross motility, spermatozoal concentration, and percent post-thaw motility, collected over a 20-year period was used to estimate genetic parameters in sheep. Univariate and bivariate mixed model analysis was performed including a variety of fixed effects such as breed, age at collection, centre of collection, collection number, season of collection, and method of collection; and the permanent environmental effects associated with each ram, stud and year of collection, and the breeding value of rams included as random effects. The heritability for ejaculate volume, gross motility, concentration, and percent post-thaw motility was estimated to be 0.161, 0.170, 0.089, and 0.081. Repeatability estimates were moderate, ranging between 0.4126 and 0.5265. Overall, results indicate that semen traits are lowly heritable and moderately repeatable, indicating that these traits are significantly influenced by environmental variables.

## 1. Introduction

Reproductive performance in livestock depends on several factors, from conception success through to offspring survival, and is crucial for overall farm profitability, by ensuring constant production of offspring for economic gain, replenishment of flock numbers, and increased genetic gain following selective breeding [1]. Despite the importance of reproductive efficiency, ejaculate traits including total motility, spermatozoal concentration and volume in livestock are reported to have declined in some longitudinal studies across 30- [2] and 17-year periods [3]. Furthermore, selection for production traits such as milk yield can indirectly result in reduced fertility on the female side in both dairy cattle [4] and sheep [5]; thus, high selection pressures on production traits may similarly result in declines in ram fertility. Given conception outcomes are positively correlated with gross motility [6], spermatozoal concentration [7], and post-thaw motility [8], ejaculate quality could potentially contribute to successful conception. Moreover, ejaculate volume and concentration influence the quantity of insemination doses produced from an ejaculate [9], and thus may influence the profitability of seedstock production enterprises involved in the sale of semen doses used in artificial breeding. Furthermore, ejaculate volume is genetically correlated (0.18–0.21) with first litter size in gilts [10], and ejaculate motility and morphology are known to influence twinning rate in humans [11]. Semen traits could similarly play a role in influencing litter size in livestock species, including sheep which, in turn, may influence the profitability of sheep enterprises. Given maiden ewes have significant abortion and lamb mortality rates compared to multiparous ewes [12,13], improving semen quality and quantity traits through selective breeding may also afford opportunities to improve maiden ewe reproduction rates. As such, every ejaculate collected should be routinely assessed to determine quality prior to use in artificial breeding programs. Moreover, estimation of heritability and genetic correlations of semen quality and quantity traits could be advantageous, and potentially inform any future efforts to selectively breed for improvement of semen traits in the Australian sheep industry.

Historically, greater emphasis has been placed on estimating the heritability associated with production and maternal reproductive traits in different populations [14,15,16,17], with relatively few studies focused on estimating the heritability for semen quality traits, particularly in sheep breeds common in Australia. The few studies that do exist indicate that heritability of semen traits is generally low to moderate. For example, a study on Spanish dairy rams estimated the heritability for common ejaculate quality traits including volume, concentration and motility to range from 0.077 to 0.304 using a dataset consisting of ejaculates from 3289 rams across five breeds [18]. Similarly, a study focused on 9- and 12-month old Ethiopian rams estimated the heritability for ejaculate volume to be 0.07 and 0.11, for gross motility to be 0.32 and 0.27, for individual motility to be 0.32 and 0.16, and for concentration to be NA and 0.17 respectively [19]. Similar low to moderate ranges of heritability estimates have been previously reported for ejaculate quality traits across several livestock species including goats [20], cattle [21,22,23], and pigs [10,24,25].

Several studies in livestock species have also estimated the genetic correlation between different combinations of semen traits [10,18,19,20,21,22,23,24,25]. Specifically, in sheep, previous studies vary considerably in terms of the reported estimates for genetic correlations between different traits. The correlation between gross motility and concentration, for example, has been estimated to be as high as 0.98 in 9-month-old rams [19], and as low as −0.04, in rams of Latxa Cara Negra breed [18]. Similarly, the correlation estimates between ejaculate volume and gross motility has been reported to vary from 0.51 in 12-month rams [19] to −0.37 in Latxa Cara Rubia [18], and the correlation between volume and concentration has been reported to vary between −0.71 and 0.02 in Latxa Cara Rubia and Churra rams respectively [18].

Spermatogenesis and semen quality in rams is known to be influenced by several factors including; age [26], nutrition [27], time of year [28], management and environment [29], method of collection [30], and potentially spermatozoal transcripts [31]. As such, it is important to account for these variables in the estimation of genetic parameters to improve the accuracy of estimates. Therefore, the objective of this study was to estimate genetic parameters associated with four key semen traits in sheep breeds commonly found in Australia, while accounting for significant environmental factors which may influence these traits.

## 2. Materials and Methods

### 2.1. Ejaculate Quality Data

#### 2.1.1. Retrospective Semen Data

Retrospective data on ejaculate quantity and quality phenotypes were provided by a commercial artificial breeding centre in Australia. Data was collected over a 20-year period from November 2000 to May 2021. These records were collected across two artificial breeding centres in the Central West and New England areas of New South Wales. The dataset included a total of 20,114 ejaculate records collected across 1724 individual rams from 17 breeds. All rams included in this dataset were transported to the commercial artificial breeding centre from their respective owner’s flock locations, and hence environment, health, and management associated with these rams was variable.

#### 2.1.2. Semen Quality Assessment

Semen collection and analysis were performed by the commercial artificial breeding centre as per standardised procedures, which are largely consistent with widely used protocols [9]. The commercial artificial breeding centre discarded an ejaculate if it did not meet the centre’s quality control (QC) standards. For example, ejaculates with a gross motility score less than three were discarded and were not subsequently cryopreserved. Therefore, some traits, such as concentration and percent post-thaw motility, were not available for all ejaculate records. Briefly, ejaculates were collected by two methods; via artificial vagina (AV), which represented the method of collection for most ejaculates in the dataset, and via electro-ejaculation [30,32]. The artificial breeding centre recorded collection number if multiple ejaculates were collected from a ram in a single day, and age of the ram (in years) was recorded on entry into the artificial breeding centre. Phenotype data recorded by the artificial breeding centre included ejaculate volume, gross motility, concentration, and percent post-thaw motility. Ejaculate volume records were produced by visual assessment of semen (in millilitres; mL), in a graduated collection vial. Gross motility scores were produced by the artificial breeding centre via microscopic examination (Olympus CH 30 with warm stage, Tokyo, Japan) of a drop of raw semen placed on a pre-warmed slide, and subsequently scoring the ejaculate based on the intensity of wave motion on a five point scale [9]. The artificial breeding centre also assessed ejaculate concentration in units of million spermatozoa per millilitre (x10^6^/mL) using a spectrophotometer (SDM1, Minitube, Germany). Widely used standardised procedures were used for cryopreservation [9]. Subsequently, progressive percent post-thaw motility was visually determined by trained staff via microscopic analysis, as described in previous studies [19,33].

### 2.2. Quality Control

#### 2.2.1. Quality Control

Prior to the descriptive statistical analysis and genetic covariance estimation, quality control was performed. Briefly, breeds with ejaculate records for less than 30 individual rams were removed. Rams were removed from analysis if their location of origin did not meet a threshold of 300 ejaculate records. Furthermore, only individual rams with pedigree data were included. Following quality control, a total of 11,470 ejaculate records were assessed from 864 individual rams across 5 breeds, including Dohne Merino (*n* = 150), Dorper (*n* = 31), Merino (*n* = 574), Poll Dorset (*n* = 65), and White Suffolk (*n* = 44) (Table 1). Pedigree data for the 864 rams was provided by Sheep Genetics (Meat and Livestock Australia).

Initial descriptive statistical analysis was performed using R software package, version 4.1.2 [34], with data in Table 1 representing means and standard deviation of untransformed ejaculate quality traits studied following data quality control. The mean of the ejaculate quality traits included in this study are in line with previous reports [9,18].

#### 2.2.2. Transformation

On preliminary analysis of volume, gross motility, concentration, and percent post-thaw motility, the residuals were found to be moderately or severely skewed. As such, the data was transformed to remove skewness in the distribution of residuals to the extent that was possible. A square root transformation was used for both volume and concentration due to positively skewed residuals (sqrt(x)), a modified square root transformation was used for percent post-thaw motility due to moderately negatively skewed residuals (sqrt(max(x + 1) − x)), and an inverse transformation was used for gross motility due to severely negatively skewed residuals (1/(max(x + 1) − x)). Supplementary Figure S1 shows distribution of residuals obtained after final mixed model analysis of transformed ejaculate quality traits.

### 2.3. Estimation of Genetic Parameters

The statistical significance of fixed effects was determined via linear regression analysis in R, to determine whether fixed effects of breed (*n* = 5), age at collection (*n* = 10), collection centre (*n* = 2), collection number (covariate), season (*n* = 4), and method of collection (*n* = 2) were to be included as fixed effects in the estimation of genetic parameters. The fixed effects included in the final analysis for each ejaculate trait are presented in Supplementary Table S1.

WOMBAT software [35] was used to estimate heritability and repeatability of the transformed ejaculate quality traits with a REML analysis. Univariate analysis of ejaculate quality traits involved mixed model regression using a number of fixed effects and covariates (specified in Supplementary Table S1), a random polygenic effect (numerator relationship matrix), a random effect associated with stud-year, and a random effect associated with residuals. Season was included in the analysis as a fitted effect to account for differences associated with seasonality that are known to influence ovine reproduction. Simply, we defined season to include 4 factors which represented peak and low reproductive season, as well as a factor between each to account for gradual differences in seasonality and photoperiod influencing sheep reproduction. Similarly, stud-year was formulated as a combination of the stud of breeding and year of collection, to account for differences between individual farms including geographical influences, management styles, nutritional profiles, and flock health status.

The full model fitted for each ejaculate trait including significant fixed effects (Supplementary Table S1) was as follows;

y = Xb + Z_1_a +Z_2_c +Z_3_d + e

where y represents the vector of transformed phenotypes for individual ejaculate quality traits; X, Z_1_, Z_2_ and Z_3_ are design matrices; b represents a vector of fixed effects; a represents a vector of random polygenic effects; c represents a vector of random permanent environmental effects; d represents a vector of random stud-year effects; and e represents a vector of random residuals.

Estimates of repeatability (r) were calculated as follows;

$$r=\frac{\sigma_{a}{}^{2}+\sigma_{c}{}^{2}+\sigma_{d}{}^{2}}{\sigma_{pe}{}^{2}}$$

where σ_a_^2^ is the estimate for additive genetic variance and σ_c_^2^ is the variance estimate for random permanent environmental genetic effects; σ_d_^2^ is the estimate of random stud-year effect; and σ_pe_^2^ is the phenotypic variance (being the sum of additive genetic, random permanent environmental, stud-year and residual variance effects). Following model fits for each ejaculate trait, bivariate analysis was performed using WOMBAT [35] to obtain estimates of phenotypic and genetic correlations between traits.

Bivariate analysis was estimated for all possibly combinations of traits, including significant fitted effects (Supplementary Table S1). The bivariate model is as follows;

$$\left|\begin{array}{c} y_{1}\\ y_{2} \end{array}\right|=\left|\begin{array}{cc} X_{1} & 0\\ 0 & X_{2} \end{array}\right|\;\left|\begin{array}{c} b_{1}\\ b_{2} \end{array}\right|+\left|\begin{array}{cc} Z_{a}^{1}{}_{1} & 0\\ 0 & Z_{a}^{1}{}_{2} \end{array}\right|\left|\begin{array}{c} a_{1}\\ a_{2} \end{array}\right|+\left|\begin{array}{cc} Z_{c}^{2}{}_{1} & 0\\ 0 & Z_{c}^{2}{}_{2} \end{array}\right|\left|\begin{array}{c} c_{1}\\ c_{2} \end{array}\right|+\left|\begin{array}{cc} Z_{d}^{3}{}_{1} & 0\\ 0 & Z_{d}^{3}{}_{2} \end{array}\right|\left|\begin{array}{c} d_{1}\\ d_{2} \end{array}\right|+\left|\begin{array}{c} e_{1}\\ e_{2} \end{array}\right|$$

where subscripts _1_ and _2_ represent the vector of transformed phenotypes in the genetic correlation, and the other terms have been previously described in the univariate model.

The variance–covariance structure of the random effects (animal, stud-year, and permeant environment) is assumed as follows;

$$V\left|\begin{array}{c} a\\ c\\ d\\ e \end{array}\right|=\left|\begin{array}{cccc} G⊗A & 0 & & \\ 0 & P\;⊗I & & \\ 0 & 0 & S\;⊗I & \\ 0 & 0 & & R\;⊗I \end{array}\right|$$

where G, P, S, and R represent the genetic covariance, permanent environment, stud-year, and residual covariance matrices respectively, A represents the numerator relationship matrix, I represent the identity matrix, ⊗ represents the Kronecker product function, and a, b, c, d, and e are defined as above.

## 3. Results

A total of 11,470 ejaculates records were assessed from 864 rams with pedigree records. Rams, selected by their respective owners as having desirable genetics, were between 1 to 10 years of age, averaging 2 and a half years old at the time of semen collection. Mean ejaculate quality traits for 11,470 semen collection records across 20-years were 1.3, 4.2, 4198 and 55.7% for volume, gross motility, concentration, and percent post-thaw motility respectively. The mean ejaculate volume ranged from 1.1 to 1.4 mL across the five breeds. Similar means for gross motility score were found across breeds, ranging from 4.1 to 4.3 out of 5. Mean values for spermatozoal concentration ranged from 3923 million sperm/mL of ejaculate for the Dorper to 4333 for the Poll Dorset. The lowest mean value for percent post-thaw motility was the White Suffolk with 49.1%, extending to the highest mean value from the Dohne with 59.7% motility following cryopreservation. Of the five assessed breeds, four had a mean post-thaw motility above 53%, with the White Suffolk being the only breed with a mean less than 50% of motile sperm following cryopreservation.

Estimates for variance, as well as repeatability estimates for volume, gross motility, concentration, and percent post-thaw motility are presented in Table 2. Estimated additive, environmental, residual, and phenotypic variances are comparable for ejaculate volume and gross motility, but the estimated variance for spermatozoal concentration is considerably higher in comparison to the other traits. Repeatability estimates for all semen traits are moderate, ranging from 0.4126 to 0.5265.

Table 3 presents the estimated heritability, genetic and phenotypic correlations for ejaculate volume, gross motility, concentration, and percent post-thaw motility. Across the five breeds, the lowest heritability was estimated for percent post-thaw motility (0.081). Spermatozoal concentration was also found to be relatively lowly heritable (0.089) compared to remaining semen traits. While volume and gross motility have moderately low estimates of heritability, of 0.161 and 0.170 respectively. Genetic and phenotypic correlations across the four ejaculate quality traits were moderate, ranging from −0.630 to 0.321 and −0.074 to 0.347 respectively. Most genetic correlations for volume were negative, with all genetic correlations for percent post-thaw motility being negative, with exclusively positive phenotypic correlations recorded for both volume and concentration.

## 4. Discussion

The aim of this study was to estimate heritability and genetic parameters for semen traits including volume, gross motility, spermatozoal concentration, and percent post-thaw motility using data collected over a twenty-year period across five common sheep breeds in Australia. Reproductive efficiency is amongst the most important factors of livestock enterprises, significantly influencing both productivity and profitability. Selection for increased productivity in cattle [4] and sheep [5] may indirectly result in reduced fertility in males and females. However, historically, breeding programs aiming to improve reproduction traits have primarily focused on ewes [14,15,16,36], with little emphasis on improving semen quality and quantity traits. However, there are multiple studies in the literature that indicate semen traits are heritable, which suggests that selective breeding can be used to improve these traits. Particularly in the context of seedstock producers, selection for spermatozoal volume or concentration may help produce a higher number of insemination doses from rams [9], which could influence profitability. Number of offspring is also correlated with semen traits such as ejaculate volume in pigs [10], and motility and morphology in humans [11]. Thus, semen quality and quantity traits may also be correlated with litter size in sheep, as such, improving semen quality and quantity traits through selective breeding may, in turn, increase the number of lambs born and, thus, overall sheep farm productivity and profitability. Moreover, since both gross and post-thaw motility are correlated with successful conception, selective breeding of these traits could improve reproductive performance of sheep [6,37]. Since knowledge about variances associated with genetic and environmental effects is a prerequisite to any efforts to selectively breed sheep, estimating the heritability and genetic parameters associated with semen traits in Australian sheep could provide insights into the potential of breeding for improved semen quality and quantity.

Ejaculate volume is one of two factors required to determine the number of insemination doses that can be produced from an ejaculate. In our results, heritability of ejaculate volume was estimated to be 0.161 ± 0.041, which is consistent with previously reported estimates [18,19,38]. One such study investigated the influence of season on several ejaculate traits for 9- and 12-month old Ethiopian highland rams, reporting heritability estimates of ejaculate volume to be 0.07 and 0.11 respectively [19]. Similarly, a multi-breed study involving Spanish dairy rams aged between 10 months to 12.5 years, found the estimated heritability of ejaculate volume to range from 0.08 to 0.20 [18]. A different study involving French rams, found the heritability of ejaculate volume to be much higher, at around 0.27 [38]. However, ejaculate records included in this study were only recorded between May and August, which may have reduced environmentally induced variability in ejaculate volumes, resulting in a higher heritability estimate. Previously published estimates of heritability of ejaculate volume in other species also align with the current study. For example, the estimated heritability of ejaculate volume has been reported to range between 0.18 [21] and 0.20 [22] for bulls, and 0.018 [10] and 0.38 [39] in pigs.

The concentration of spermatozoa in an ejaculate is the second necessary factor in the calculation of an insemination dose. Spermatozoal concentration is important, as too many spermatozoa negatively impact post-thaw motility and viability [40], and too few spermatozoa can similarly negatively influence semen quality parameters including viability and motility [41,42], as well as potentially impairing conception rates [43,44]. Spermatozoal concentration was found to be lowly heritable in this study (0.089 ± 0.051), marginally lower than heritability estimates reported in a multi-breed study involving Spanish dairy rams (0.10 to 0.19) [18], but significantly lower than those reported in goats (0.32 to 0.34) [20]. Some of this variability could be attributable to the method of analysis used to assess spermatozoal concentration, which contribute to phenotypic variance [45]. For example, some studies involved ram semen assessed with calibrated Photometers [22], or colorimeters, which require dilution of an ejaculate prior to assessment of spermatozoal concentration [20]. Other studies have used slightly different protocols of assessment such as averaging two individual ejaculates from a single sire collected 2 to 5 min apart [18]. Additionally, estimated heritability for spermatozoal concentration of Ethiopian sheep was reported at 0.17 for 12-month-old rams [19]. However, unlike this study, Rege et al., [19] estimated the heritability of semen quality traits fitting covariates associated with ewe side reproductive traits. Estimates of heritability for spermatozoal concentration in other species are variable, e.g., 0.19 for dairy cattle at a commercial artificial insemination (AI) centre [3], 0.07 to 0.34 across seven breeds of Swiss dairy bulls [46], 0.19 [47], and 0.13 to 0.23 for boars at a commercial AI centre [48].

The gross motility of ejaculates used in this study was assessed on a five-point scale, with records collected over a 20-year period. In our analysis, gross motility was estimated to have a heritability of 0.170 (±0.058). This accords with previously reported estimates of 0.12 to 0.17 for Ethiopian sheep breeds [19], and 0.01 to 0.11 across five breeds of Spanish dairy rams, with ejaculates collected across 3 AI centres [18]. Another study involving analysis of semen traits collected from Lacaune and Manech rams at two French AI centres, reported the heritability of gross motility for each breed to range between 0.07 and 0.14 respectively [49]. Previous studies in sheep have also used gross motility data skewed towards higher scores to estimate heritability and genetic parameters [18,49]. Skewed gross motility data, in both previous studies and the current study, is likely attributable to the fact that rams entering commercial breeding facilities for semen collection are generally well-managed in the context of factors which can influence semen production and quality, such as nutrition, condition score, stress. Studies in other species, have estimated that the heritability of gross motility varies considerably from 0.16 [3], 0.04 [21], and 0.37 [22] in bulls, to around 0.6 and 0.16 in two different breeds of pigs [48].

The heritability for percent post-thaw motility was low in our study (0.081 ± 0.040). This aligns with heritability estimates for total and progressive motility in pigs (0.08 and 0.10 respectively) following dilution with a cryo-diluent [50]. However, similarly sized studies involving 10 years’ worth of ejaculate records across two breeds of goat have reported higher heritability for post-thaw motility (0.12 and 0.17) [20]. Heritability estimates of percent of total motile spermatozoa immediately following ejaculation were 0.32 and 0.16 for 9- and 12-month-old Ethiopian rams [19]. Furthermore, a study involving ejaculate records collected over a 7-year period from a variety of pig breeds estimated the heritability of percent total motility of fresh ejaculates to be 0.38 [24]. Berry at al. [22] reported notable declines in heritability estimates for both percent alive spermatozoa (0.34 and 0.25) and gross motility (0.37 and 0.13) for pre- and post-cryopreservation respectively, suggesting cryopreservation significantly influences heritability estimates. It has been established that several factors associated with cryopreservation influence post-thaw motility, including cryo-diluent used [51], both cryopreservation [52] and thawing processes [53], as well as the animals’ genetics [54]. Differences in some or all factors could potentially influence percent post-thaw motility, including in our study; thus, it is not unusual to see differences in the heritability estimates of percent motility throughout previous studies. It is also important to note that, despite training and experience, if the evaluation of percent motility of and ejaculate is being performed by a technician, bias could result in a difference in percent motility from 15 to 80% [55], further accounting for the differing reports of heritability estimates in the literature. Furthermore, this study assessed ejaculate traits across a 20-year period; thus, estimates of environmental variance (0.2383) in the current study may differ to previous reports examining ejaculate traits across a smaller period [19,20,24].

Overall, studies focused on analysis of ovine ejaculate quality data have consistently reported that these traits are heritable. The variability in heritability estimates between different studies is likely attributable to differences in number of animals, breeds, records, AI centres, and period of sampling [18,19]. Despite these differences, the heritability estimates for some traits are similar in different studies (see Supplementary Table S2). For example, two independent sheep studies estimated the heritability of ejaculate volume to vary between 0.07 and 0.11 [19], and 0.08 and 0.20 [18]; and concentration to vary between 0.17 [19], and 0.10 and 0.19 [18]. Contrary to this, in these same studies, estimated heritability of gross motility varied significantly from 0.32 to 0.27 [19], and 0.01 to 0.11 [18] across different ages and breeds. Relatively fewer studies have focused on percent post-thaw motility and, in at least one study, the heritability of fresh progressive motility was estimated to vary between 0.16 and 0.32 in Ethiopian rams [19], and progressive motility following cryopreservation ranged between 0.03 and 0.05 for two breeds of goat [20]. Results from other studies focused on semen traits in cattle [22], and pigs [48] generally align with the current study. However, in at least one porcine study [24], a number of semen traits, including volume, concentration, and percent post-thaw motility, were found to be highly heritable. This is likely because permanent environmental effects influencing multiple ejaculates from the same boars were not modelled during analysis, which, in turn, can cause an upward bias in estimated additive genetic variance [56], and consequently heritability. Similarly, other studies with relatively smaller resource populations, or relatively larger sampling periods, can report lower heritability estimates of as a consequence of low additive genetic variance, or high environmental variance respectively [57].

The phenotypic and genetic correlations between semen traits in this study are in line with previous studies [18,19]. Heritability estimates for all traits except concentration were twice the standard error indicating significance. The heritability estimate for concentration was approximately 1.75 times the standard error, which may be due to fewer records available for this trait. Estimates for genetic correlations, in contrast, were mostly non-significant, except for the correlation between gross motility and percent post-thaw motility. The high standard errors associated with correlation estimates have been reported in sheep [18], which may be due to considerable environmental variance associated with these phenotypes. Positive genetic and phenotypic correlations between gross motility and concentration were previously reported in Ethiopian [19] and Spanish dairy sheep [18]. Moreover, the negative genetic correlation between ejaculate volume and gross motility aligns with previous studies in Spanish dairy sheep [18]. It is important to note that, although the negative correlation between gross and post-thaw motility in this study appear to stand in contrast to others studies, where a positive correlation has been reported [3,47], this is due to the square root transformation applied to percent post-thaw motility, which reverses the ranking of phenotypes. Taking this into account, the correlation between gross and post-thaw motility in this study is, therefore, positive and consistent with previous studies. Data transformation to address non-normal distribution of residuals has previously been reported in semen-quality studies in different animal species such as bulls [58], and horses [59]. Studies have also reported the transformation of semen phenotypes does not impact the magnitude of estimated heritability and genetic parameters [60]. Furthermore, low phenotypic correlations between semen motility traits in the current study align with past estimates [18,19], suggesting that environmental influences, such as handling of semen by AI technicians, may have a significant impact on estimating covariance of ejaculate motility traits. Additionally, given ejaculate quality and quantity are influenced by several genetic and non-genetic factors, care must be taken when estimating heritability and genetic parameters, and further when using heritability estimates to determine breeding values, which may assist in selection of sires based on ejaculate quality and quantity.

## 5. Conclusions

In conclusion, heritability estimates of semen traits such as ejaculate volume, gross motility, concentration, and percent post-thaw motility were found to be low or moderate (0.081–0.170). This indicates that environmental variance significantly contributed to phenotypic variance of these traits, which is consistent with the fact that the data used in this study was collected over an extensive 20-year period. Regardless, these results also indicate that variability in semen traits is, at least in part, determined by genetics, which indicates that these traits could potentially be improved via selective breeding. However, given the relatively low heritabilities, and the potential for that some of these traits may be correlated with other economically important traits, functional studies focused on identifying genes and biological mechanisms underlying these traits may be warranted. Future studies to establish whether semen traits are correlated with other economically important production traits are also warranted.

## Figures and Tables

**Table 1:** Descriptive statistics for ejaculate quality traits analysed for each breed.

Breed	Number of Rams	Number of Records	Mean	Standard Deviation
Volume (mL)				
Dohne Merino	150	1946	1.4	0.6
Dorper	31	320	1.2	0.5
Merino	574	8130	1.3	0.5
Poll Dorset	65	595	1.1	0.4
White Suffolk	44	479	1.1	0.4
Gross Motility (0–5)				
Dohne Merino	150	1925	4.1	1.2
Dorper	31	313	4.2	1.0
Merino	574	8055	4.2	1.2
Poll Dorset	65	549	4.2	1.2
White Suffolk	44	468	4.3	1.0
Concentration (×10^6^/mL)				
Dohne Merino	143	1541	4099	1428
Dorper	30	255	3923	1162
Merino	555	6618	4233	1133
Poll Dorset	63	420	4333	1043
White Suffolk	42	383	4038	867
Percent Post-Thaw Motility (%)				
Dohne Merino	133	1235	59.7	13.9
Dorper	30	179	56.2	15.6
Merino	541	5506	55.2	14.9
Poll Dorset	62	330	53.2	18.1
White Suffolk	42	285	49.1	17.9

**Table 2:** Genetic parameter estimates for the four transformed ejaculate traits studied, volume, gross motility, concentration, and percent post-thaw motility.

	σ_a_^2^	σ_c_^2^	σ_d_^2^	σ_e_^2^	σ_pe_^2^	R (±SE)
Volume	0.0086	0.0064	0.0070	0.0313	0.0533	0.4126 (0.0154)
Gross Motility	0.0152	0.0187	0.0132	0.0424	0.0895	0.5265 (0.0146)
Concentration	8.6941	21.7847	13.6542	53.8614	97.9944	0.4504 (0.0165)
Percent Post-Thaw Motility	0.1096	0.2383	0.2729	0.7371	1.3579	0.4572 (0.0176)

Note: σ_a_^2^: additive genetic variance; σ_c_^2^: permanent environmental variance; σ_d_^2^ variance explained by stud-year; σ_e_^2^: residual variance; σ_pe_^2^: phenotypic variance; and R (±SE): repeatability ± standard error.

**Table 3:** Genetic parameters of semen traits. Heritabilities of the transformed ejaculate quality and quantity traits are presented in bold face on the diagonal of the table. Genetic and phenotypic correlations between different combination of traits are presented above and below the diagonal respectively. Standard errors are presented in parenthesis.

	Volume	Gross Motility	Concentration	Percent Post-Thaw Motility
Volume	**0.161 (0.041)**	−0.071 (0.206)	0.153 (0.227)	−0.262 (0.274)
Gross Motility	0.215 (0.020)	**0.170 (0.058)**	0.321 (0.282)	−0.630 (0.238)
Concentration	0.228 (0.022)	0.347 (0.019)	**0.089 (0.051)**	−0.351 (0.286)
Percent Post-Thaw Motility	0.113 (0.024)	−0.074 (0.024)	0.100 (0.024)	**0.081 (0.040)**

Heritabilities of the transformed ejaculate quality and quantity traits are presented in bold face on the diagonal of the table.

## Data Availability

The data present in this study are available on request from the corresponding author.

## Supplementary Materials

The following supporting information can be downloaded at: https://www.mdpi.com/article/10.3390/ani12212946/s1, Figure S1: Histograms displaying the skew of data of residuals from WOMBAT for transformed values for four ejaculate quality traits; (A) volume, (B) gross motility, (C) concentration, (D) percent post that motility; Table S1: Significance of fitted covariates for each ejaculate trait; Table S2; Across study comparison of previously published estimates of heritability and corresponding repeatability for ejaculate traits including volume, gross motility, concentration, and percent post-thaw motility.
