# Supplementary material for: Heritability and Genetic Parameters for Semen Traits in Australian Sheep
Source: Animals (Basel). 2022 Oct 26;12(21):2946. doi: 10.3390/ani12212946 (PMC9658395; doi:10.3390/ani12212946)
Supplement: Supplementary file 1 [file animals-12-02946-s001.zip › animals-1918124-supplementary.pdf]

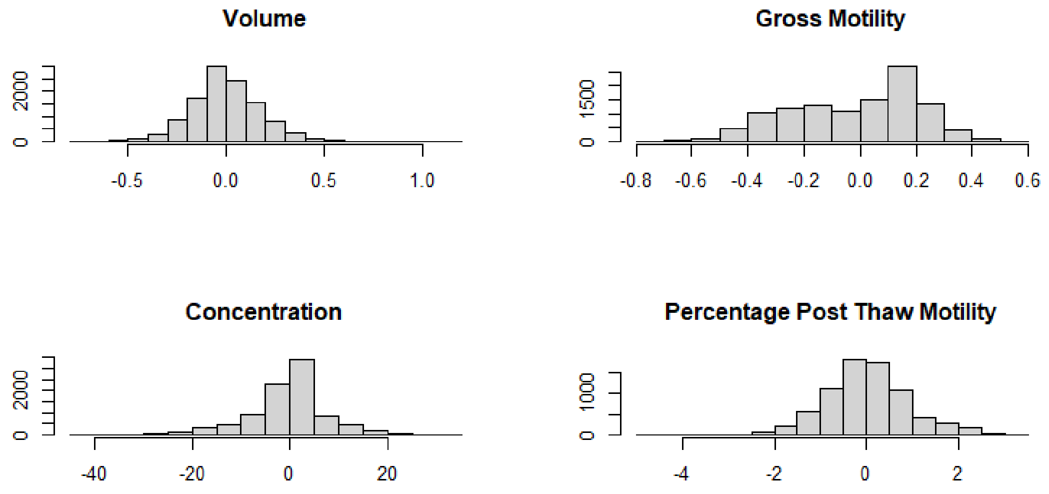

Supplementary Figure S1. Histograms displaying the skew of data of residuals from WOMBAT for transformed values for four ejaculate quality traits; A) volume, B) gross motility, C) concentration, D) percentage post that motility.

Supplementary Table S1. Significance of fitted covariates for each ejaculate trait

	Volume	Gross Motility	Concentration	Percentage Post Thaw Motility
Breed	***	**	***	***
Age at collection	***	***	***	***
Collection centre	***	***	***	NS
Collection number	***	NS	***	***
Season	***	***	***	***
Collection method	***	NS	NS	NS

Note: *** = (p < 0.001), ** = (p < 0.01), * = (p < 0.05), and NS = (p > 0.05)

Supplementary Table S2. Across study comparison of previously published estimates of heritability and corresponding repeatability for ejaculate quality traits including volume, gross motility, concentration, and percentage post thaw motility

Species	Number of Animals	Number of Breeds	Number of Records	Sampling Period (years)	Number of AI Centres	Heritability Estimate	Repeatability	Reference
Volume (ml)								
Sheep	483	2	483 (2 age groups)	1992 – 1993	1	0.07 – 0.11	NA	[14]

Sheep	3,289	5	96,790	2003 – 2009	2	0.08 – 0.20	0.21 – 0.30	[13]
Cattle	787	16	35,573	2000 - 2015	1	0.02	0.33	[18]
Pigs	2,862	19	210,733	1990 - 1997	31	0.58	NA	[19]
Pigs	3,675	14	215,830	2000 – 2005	23	0.14 – 0.25	0.43 – 0.46	[35]
Gross Motility (0-5)								
Sheep	483	2	483 (2 age groups)	1992 – 1993	1	0.32 – 0.27	NA	[14]
Sheep	3,289	5	96,790	2003 – 2009	2	0.01 – 0.11	0.08 – 0.20	[13]
Cattle	787	16	31,585	2000 - 2015	1	0.37	0.38	[18]
Pigs	2,862	19	210,733	1990 - 1997	31	NA	NA	[19]
Pigs	3,675	14	215,830	2000 – 2005	23	NA	NA	[35]
Concentration (x10^{6}/mL)								
Sheep	483	2	483 (2 age groups)	1992 – 1993	1	NA – 0.17	NA	[14]
Sheep	3,289	5	96,790	2003 – 2009	2	0.10 – 0.19	0.27 – 0.30	[13]
Cattle	787	16	35,074	2000 - 2015	1	0.02	0.45	[18]
Pigs	2,862	19	210,733	1990 - 1997	31	0.49	NA	[19]
Pigs	3,675	14	215,830	2000 – 2005	23	0.13 – 0.23	0.37 – 0.38	[35]
Percentage Post Thaw Motility (%)								
Sheep	483	2	483 (2 age groups)	1992 – 1993	1	0.32 – 0.16	NA	[14]
Sheep	3,289	5	96,790	2003 – 2009	2	NA	NA	[13]
Cattle	787	16	23,614	2000 - 2015	1	0.25	0.42	[18]
Pigs	2,862	19	210,733	1990 - 1997	31	0.38	NA	[19]
Pigs	3,675	14	215,830	2000 – 2005	23	0.06 – 0.16	0.29 – 0.35	[35]
